# The effect of exercise-induced fatigue and heat exposure on soccer-specific decision-making during high-intensity intermittent exercise

**DOI:** 10.1371/journal.pone.0279109

**Published:** 2022-12-15

**Authors:** Kate J. Donnan, Emily L. Williams, Nicholas Stanger

**Affiliations:** 1 Department of Sport, Health and Exercise Science, University of Hull, Hull, United Kingdom; 2 Institute for Sport, Physical Activity and Leisure, Leeds Beckett University, Leeds, United Kingdom; Federal University of Paraiba, BRAZIL

## Abstract

Global warming and the globalisation of sport has increased the prevalence of sports competitions being held in hot environments. However, there is currently limited research investigating the impact of the heat on soccer-specific decision-making skills during exercise reflective of the physical demands of match-play. Therefore, the effects of heat exposure on physical and soccer-specific decision-making performance, biological markers (i.e., metanephrines), appraisal (i.e., challenge vs. threat) and affective states, during prolonged high-intensity intermittent exercise were investigated. Nine well-trained male soccer players completed a 92-min cycling intermittent sprint protocol (CISP), whilst simultaneously responding to a series of soccer-specific decision-making trials at various time points, in two temperature conditions: hot (32°C, 50%rh) and temperate (18°C, 50%rh). Results showed that decision-making score (*p* = .030) was impaired in the hot compared to the temperate condition. There was a reduced workload in the second half during the hot condition (*p* = .016), which coincided with a heightened threat state (*p* = .007) and more unpleasant feelings (*p* = .008) experienced in the hot, compared to temperate, condition. Furthermore, plasma normetanephrine (NMET) was higher at half-time (*p* = .012) and post-CISP (*p* ≤ .001). Also, plasma metanephrine (MET) was higher post-CISP (*p* = .009) in the hot compared to temperate condition, reflecting a heightened stress response. Our findings highlight the need for practitioners to consider the detrimental effects heat exposure can have on both physical and decision-making performance when looking to facilitate performance in hot conditions.

## Introduction

Successful performance in soccer is dependent upon the simultaneous execution of a range of physical and cognitive skills. Sport-specific perceptual abilities can distinguish between elite and novice players [e.g., 1,2], with skilled athletes known to anticipate future events and search viable options beyond the next move [3,4]. Effective decision-making is described as the perception and correct interpretation of information from the environment, and the selection of an appropriate response [5], which encompasses two key factors, processing speed and accuracy [6]. Although effective decision-making and sporting intelligence will aid a soccer player in making correct decisions from a variety of stimuli (e.g., identification of space, predicting opposition movement and receiving/giving instructions), the execution of such cognitive skills is also dependent upon other factors whilst competing, including the onset of fatigue [7].

During the physically demanding nature of competitive soccer, physical fatigue occurs due to a depletion in energy stores (i.e., glycogen), increased thermal strain, dehydration, and the alteration of central mechanisms (e.g., neurochemical change) [8]. Soccer players cover less distance [9] and skilled performance has been shown to deteriorate in the second half, compared to the first half of match-play [10]. However, fatigue has also been associated with altered perceptual states and impaired central nervous system functioning [11] that can impact decision-making ability.

Research in team sport has shown that high-intensity or prolonged exercise can improve decision-making speed [12,13], but may impair decision-making accuracy or quality [e.g., 13,14]. These effects have been linked to variations in arousal [15] induced by physiological changes (e.g., plasma catecholamines, cortical activation) at central and peripheral levels [16]. That said, some reviews have highlighted ambiguous findings whereby no effects or positive effects of high-intensity or prolonged exercise on cognitive function have been found [e.g., 17–19]. Such variations in findings may be explained by differences in methodologies such as the timing of cognitive function. Based on premises of the ‘transient hypofrontality hypothesis’, during demanding physical work, neural resources are re-directed away from pre-frontal areas of the brain responsible for higher-order (or more complex) cognitive functioning, to areas of the brain responsible for maintaining motor control to prioritise maintenance of physical work. [20]. However, when exercise has abated neural resources are suggested to be re-directed back toward pre-frontal areas [20]. Thus, differences in methodologies such as the timing and complexity of cognitive task implemented (i.e., pre- to post- exercise, or during) can influence the effects that exercise can have cognitive function, which could explain some of the varied observations in previous research. Additionally, the decision-making tasks employed in most studies to date are not situation-specific, thus they can be more predictable than decisions faced within match-play and have limited ecological validity. Therefore, research investigating the effects of incremental fatigue on sport-specific decision-making during exercise warrants further attention.

As well as the typical incremental fatigue experienced during competitive soccer matches in thermoneutral environments, athletes can be required to perform in hot environments, such as during pre-season and international tournaments [e.g., 2022 Qatar FIFA World Cup, 21]. Performing in high environmental temperatures can exacerbate the physiological and perceptual strain experienced during match-play such as increased core temperature (T_C_), cardiovascular strain (e.g., increased heart rate), dehydration, and neurochemical alterations [22,23], resulting in physical [24] and cognitive [e.g., 25,26] performance decrements.

Previous research has also revealed that physiological load or the incremental fatigue experienced during exercise could also disrupt one’s attention during sporting performance. For instance, Casanova et al. [27] found that increased physiological loads caused soccer players’ attention to be directed to task-irrelevant cues more often, resulting in reduced anticipation accuracy. ‘Think-aloud’ retrospective reports exploring how expert players used situational information when making anticipation judgments, showed that deep planning statements decreased from early in the first half, to the end of the first half and second half of soccer-specific activity. This reflected a deterioration in higher level thought processing with fatigue, which may be further impaired under additional physiological strain induced by heat exposure. However, research employing such ‘think aloud’ methods to understand athletes’ reasoning for decisions as a result of incremental fatigue is limited. Indeed, researchers have yet to examine the effect that heat exposure can have on such thought processes in athletes during exercise that is reflective of the physiological load experienced during match-play.

It is possible that heat stress and incremental fatigue can also impact an athlete’s appraisal of the activity demands, and previous research has revealed that it can result in more unpleasant affective states [e.g., 14], which could also be linked with physical and cognitive performance. The Theory of Challenge and Threat States in Athletes [TCTSA; 28] offers insights into how athletic performance may be impacted by competitive stress and the appraisal of an activity. It is suggested that a challenge state is usually (but not always) associated with positively valenced emotions as well as linked with effective decision-making, higher task engagement, and positive performance. In contrast, a threat state is suggested to be associated with (typically) negatively valenced emotions as well as emotions that are perceived as unhelpful for performance, impaired cognitive functioning, reduced task engagement and performance decrements [28].

The TCTSA also identifies physiological responses associated with the appraisal of an activity. Increased sympathetic-adrenal medulla (SAM) activity is proposed to represent a challenge state, whereas a threat state is posited to be characterised by increased SAM and pituitary-adrenal cortical (PAC) activity, both of which are affected by heat exposure. Specifically, acute heat stress has been shown to increase both SAM activity (e.g., increased release of epinephrine and norepinephrine) and PAC activity (e.g., increased adrenocorticotropic hormone and cortisol release) [29]. Therefore, where SAM and PAC activity increase in response to acute heat stress [29], performing strenuous activity in hot environments may also contribute to athletes’ appraising activity as more threatening compared to temperate conditions [28]. To the authors’ knowledge, only one study has tested and found that heat exposure during intermittent exercise can make athletes feel more unpleasant compared to temperate conditions [14], and researchers have yet to directly investigate whether heat exposure could also influence athletes’ appraisal during prolonged high-intensity intermittent exercise. Such examination would offer novel insights about how heat exposure can influence appraisal and affective states during exercise in the heat, which could help inform approaches to assist athletes coping when performing in hot conditions.

### Purpose of the research

To date, no research has investigated the effects of prolonged intermittent exercise and heat exposure on soccer players’ decision-making. Moreover, through the implementation of ‘think-aloud’ protocols [e.g., 27], it is known that fatigue can impair higher level thought processing in soccer players. However, to date, no study has yet to explore how the additional stress imposed by heat exposure may impact such processes. Additionally, research has yet to investigate how heat exposure during prolonged intermittent exercise reflective of physical demands of match-play can affect a combination of biological (i.e., epinephrine and norepinephrine) and psychological (i.e., changes to affect, appraisal) responses that could have a role to play on performance (e.g., 28) in soccer players.

Therefore, the aim of this research was to examine how heat exposure (32˚C) during prolonged intermittent sprint exercise affected physical work output, soccer-specific decision-making, demand and resource appraisals (i.e., challenge and threat), affective valence (i.e., positive or negative feelings), and plasma metanephrines (i.e., plasma metanephrine and normetanephrine, as serum metabolite indicators of epinephrine and norepinephrine, respectively), in comparison to a temperate condition (18˚C). As it was expected that soccer players would be under greater physiological and psychological strain in the heat, the following hypotheses were tested. First, it was hypothesised that power output (measure of physical output) and the quality of decision-making would deteriorate during the later stages of the protocol as a result of exercise-induced fatigue, and that this deterioration would be more pronounced in 32˚C compared to 18˚C (H_1_). Second, it was anticipated that during exercise in 32˚C, players’ ability to engage in higher level thought processes would be reduced when retrospectively evaluating their decision, compared to during exercise at 18˚C (H_2_). Third, it was expected that threat appraisal about the activity demands, and unpleasant affect, would be higher in 32˚C compared to 18˚C (H_3_). Finally, it was believed that metanephrine and normetanephrine levels would increase in response to exercise, but more significantly in the hot, compared to the temperate conditions, reflecting a heightened physiological stress response (H_4_).

## Materials and methods

### Participants

Nine well-trained male soccer players at a university in the United Kingdom (M ± SD age = 19.89 ± 0.78 years; body fat = 11.94 ± 0.04%) with an average of 13.11 (± 1.45) years of experience competing in soccer were recruited. The highest competition standard ranged across national (*n* = 2), regional (*n* = 3) and club (*n* = 4) levels. An estimated sample size of nine participants was determined by a power calculation using G*Power 3.1.9.4 [30], applying an estimated large effect (i.e., Cohen’s *d* = 1.1; *f* = 0.55) with power (1- β = .80) and significance (alpha = .05). The effect size coefficient applied was based on the results from a study which examined the effects of exercise on cognition between hot (36°C) and temperate (20°C) conditions, which found such (large) effects and significant differences for changes in cognitive performance (random movement generation) from pre-exercise to immediately post-exercise between temperature conditions in a sample of 8 participants [31].

Participants were asked to refrain from ergogenic aids for the duration of the study and should not have performed strenuous exercise in >30°C for at least 3 months prior [32]. Participants were non-smokers, with no history of cardiovascular or respiratory problems. They were asked to standardize their food intake on the morning of each trial, abstain from alcohol and caffeine for ≥24 hours, refrain from strenuous exercise for ≥48 hours, and maintain their regular sleep pattern for ≥72 hours [32]. A food (including photographs) and sleep diary was completed by each participant in the 48-hours leading to each trial to ensure standardisation was met. Participants arrived ≥2 hours post-prandial and consumed between 2–3 litres of water in the 24 hours leading to testing. Following approval from Leeds Beckett University research ethics committee, all eligible participants provided written consent before commencing testing.

### Experimental design and procedure

In total, participants visited the laboratory on five occasions over a 28-day period. The first visit was for familiarisation and the remaining four trials were separated into two parts for different purposes. Specifically, experimental trials 1 and 2 (Part A) were used to explore the psychological, biological, and decision-making implications of exercising in hot compared to temperate conditions, whereas trials 3 and 4 (Part B) were used to explore the effectiveness of Tyrosine supplementation for performing in hot conditions. Due to the extensiveness of the research undertaken, this article focuses only on Part A. Thus, a 2 × condition within-subjects design was employed whereby participants completed trials in a temperate (18°C; 50% relative humidity [rh]) and hot (32°C; 50% rh) condition. With the aim of using a fully counterbalanced design, we intended to include 10 participants. However, due to the global pandemic, testing was terminated early. Therefore, 5 participants completed their first trial in 32˚C, whilst 4 completed their first trial in 18˚C. Results for 8 participants when conditions were fully counterbalanced can be found in the Support Information. Trial days were kept ≥2 to ≤7 days apart and testing times remained consistent to control for circadian variation and subsequent effects on core temperature and power output [33,34].

During the first visit, participants completed an incremental exercise test on a cycle ergometer to determine power output at VO_2max_ [35]. Once recovered, participants were instructed to watch 10 soccer-specific clips and respond verbally as to what they would do next if they were in possession of the ball and provide reasoning for their decision. This was to familiarise participants with a newly validated decision-making protocol (detailed below). Participants were then familiarised with the cannulation process before completing a standardised warm-up, followed by 10 × 5-s sprints of the Cycling Intermittent Sprint Protocol (CISP) (20 min) to familiarise with the protocol. Simultaneous decision-making tasks (and verbal reports) were completed following every other sprint during this process. Participants then spent a short period of time (~5-min) sat in the chamber at ∼30°C to minimise any potential negative emotional reactions to the heat during trials.

The next laboratory visits comprised of the experimental conditions. For each visit, participants were provided with food vouchers, allowing them to replicate what they consumed for breakfast and lunch on the first trial day on all subsequent visits. Ad libitum water intake was permitted between the 22^nd^-28^th-^min, and between the 62^nd^-68^th^-min of the CISP, to replicate water breaks during major soccer competitions held in >30˚C [36], and total consumption (ml) was recorded. To facilitate a competitive element and incentivise effort during the trials, a financial incentive was included [e.g., 37]. Participants were informed that the player who had the highest combined average decision-making score as well as physical performance across trials would be awarded a £100 cash prize.

#### Maximal oxygen uptake (VO_2max_)

To determine power at VO_2max_, during the familiarisation visit participants were instructed to cycle at a pedal rate between 70–90 rpm. One minute at 75W was performed, after which work rate increased at a rate of 25W.min^-1^ until participants cadence declined by >10 rpm or through volitional exhaustion. Breath-by-breath pulmonary gas exchange was collected throughout (Cortex, Metalyzer 3B) and data was averaged across 10-s periods. The VO_2max_ was identified as the highest 30-s mean value attained before the point of volitional exhaustion [e.g., 38]. The power output at the point of volitional exhaustion was then used to inform the protocol in the main trials (see details below). Heart rate (HR) was measured throughout via a Polar HR monitor (FT1, Polar, Finland) and subsequently averaged for each incremental stage.

#### Cycling intermittent sprint protocol (CISP)

**Fig 1:**
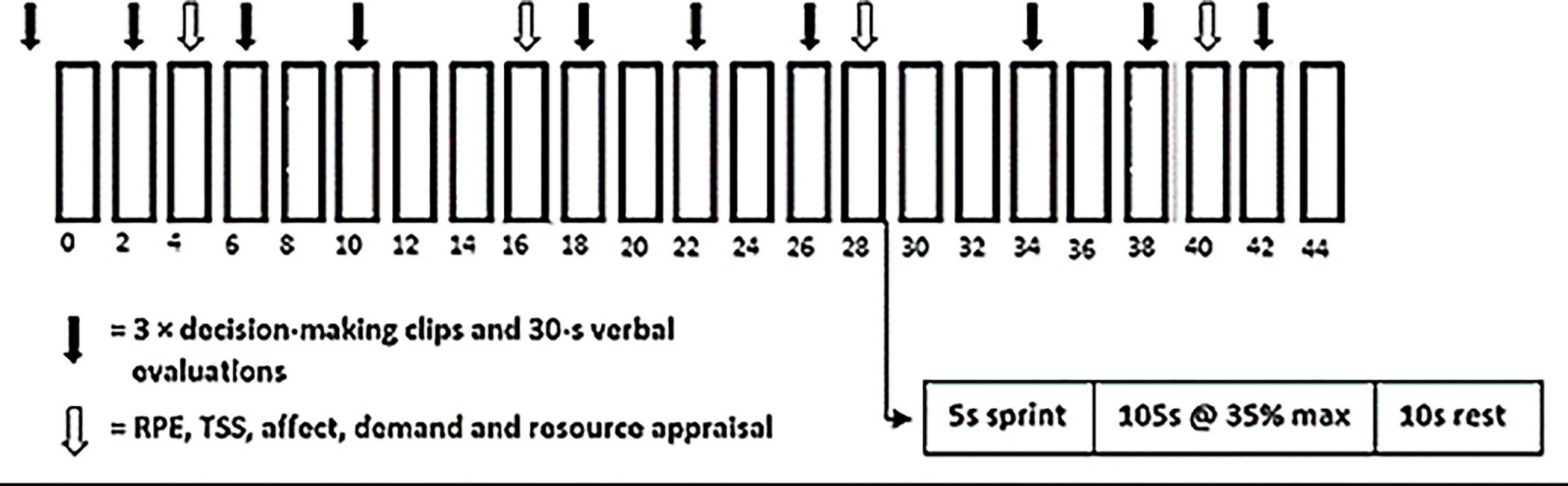
A schematic showing a modified version of one half of the extended Cycling Intermittent Sprint Protocol [39,40].

An extended version of the CISP [39] (see Fig 1) was used whereby it was increased to 46-min (23 sprints) from the original 40-min (20 sprints), per half, to better reflect the duration of soccer matches. Each trial consisted of a warm-up of 5-min cycling at 95W at 80 rpm, followed by two 30-s periods of passive rest interspersed with 30-s cycling at 120W [40]. The extended CISP consisted of 23 blocks of 2-min periods in each half consisting of a 5-s maximal sprint against a resistance of 8% body mass (BM), 105-s of active recovery at 35% VO_2peak_ calculated from the familiarisation visit, followed by 10-s passive rest [39]. Each half of the CISP was interspersed with a 15-min “half-time” period involving passive recovery in a temperate environment (~18°C). Therefore, each trial comprised of a total of 46 blocks of 2-min to simulate the demands of a typical competitive soccer match [41].

### Main outcome measures

#### Peak power output (PPO)

PPO (W) was determined as the highest power output recorded during each sprint of the CISP [39]. Lode Ergometry Manager (Version 10, Lode, Groningen, The Netherlands) automatically recorded these outputs following every sprint and this data was then transferred to Excel (Version 2008, Microsoft). These outputs were averaged per half of the CISP to analyse differences between the first and second half of match-play.

#### Decision-making

Within the trials, decision-making was assessed by asking participants to watch video clips and decide what their next choice would be for the player in possession of the ball. Clips were cut from previous 2014 and 2018 World Cup footage using Adobe Premier Pro (Adobe Inc, California, United States). Similar to previous research [27], clips lasted for ∼5 seconds and froze just before the player in possession of the ball executed their decision (e.g., shoot). At this point, participants were asked what they would do next. Prior to implementation in the present study, the decision-making protocol was validated in a preliminary study involving an ethically approved, three-stage process.

*Development of decision-making protocol*. The first stage of the validation process aimed to test the face and content validity of the clips, which involved four well-trained adult soccer players (2 men; 2 women) reviewing a total of 205 clips to assess (a) the clarity of the clips (from 1 = *not at all clear*, to, 9 = *extremely clear*), (b) ensure that a minimum of 3 realistic response options were available (e.g., pass, cross, dribble, clear, turn, shoot), and (c) rank the difficulty of making a decision from the clip (from 1 = *very easy*, to, 9 = *very difficult*). Frequent breaks were included to reduce fatigue. This process resulted in 5 clips being removed based on an insufficient number of options.

The second stage aimed to further support the face validity of the remaining clips and develop a scoring system based on an expert panel of four UEFA qualified coaches (including an ex-international player) who independently ranked what would be the best, second best and third best option in each clip. The expert panel also rated the perceived difficulty of the decision as per stage one, and breaks were again included to reduce fatigue. Based on this process, four options remained for the decision-making; namely “maintaining possession” (e.g., drive, turn), “pass” (including crossing), “clear”, and “shoot”. Following this process, the bank of clips was cut to 84, with only those with the highest agreement scores (e.g., ≥90% score, where a minimum of three experts had rated the same best available option, with one expert rating this as the second best) remaining, alongside accounting for clip difficulty. As participants completed four CISP trials in this research (two trials to address the aims in the present study), the clips were split into 4 × sets of 21 clips of similar difficulty, distributing easy, moderate, and difficult clips evenly across sets of footage, as based on expert judgements. For each overall set, three clips were intended to be used for pre-exercise, then nine clips for each half of the CISP (i.e., 3 + 9 + 9 = 21 clips). Reliability analyses were conducted to confirm that the experts’ judgement scores were similar across the 4 sets of clips (Fleiss kappa >.80), and across the block of 3 clips (intended for pre-exercise) and 2 blocks of 9 clips (intended for each half of the CISP) within each of the four sets (Fleiss kappa > .80).

The third stage involved a sample of 14 well-trained outfield female soccer players completing the decision protocol at rest, to test that the scoring for the decision-making clips did not differ between the 4 sets of 21 clips nor between the blocks of 3 clips (for pre-exercise) and 9 clips (for each half of the CISP). Indeed, analyses revealed no set × block interactions (*F*_*6*,*78*_ = .676, *p* = .669, ηp^2^ = .049), nor differences between sets of clips (*F*_*3*,*39*_ = 1.114, *p* = .355, ηp^2^ = .079), or across blocks (*F*_*1*.*295*.*16*.*835*_ = 2.374, *p* = .138, ηp^2^ = .154). Post-hoc analysis was run using LSD as the least conservative approach for observing differences and no post-hoc differences were found between any block within sets (*p*s = .189 to .932) or between sets (*p*s = .169 to .672). Overall, supporting that the sets of clips were not different in scoring at rest, and thereby suitable to be randomly allocated for the main experimental trials to determine any differences in decision-making between conditions.

*Decision-making in the present study*. Pre-exercise decision-making was measured by participants responding to 3 decision-making clips (each lasting 5-seconds). A further 9 clips in each half of the CISP were used to assess decision-making during exercise. These were regularly presented in 3 sets of 3 clips in each half and implemented at the same respective time points for each half (see Fig 1).

After each decision-making clip, the final frame froze for 3-seconds where participants were asked to provide their verbal response as to what they would do next if they were the player in possession of the ball. Participants had four initial choices when making their decision; “pass”, “shoot”, “clear”, and “maintain possession” and were asked to verbally respond as quickly and accurately as possible. If pass was selected, participants were then asked which player they intended to pass to by identifying the colour of the circle around the chosen player. Based on the rankings from experts in the preliminary study, participants were awarded 3 points for the best option, 2 points for the second-best option, and 1 point for any other response given from any of the expert panel during the validation process. Therefore, 9 points were available pre-CISP (from the 3 clips), and a maximum of 27 points were available for each half of the CISP (from the 9 clips). For analysis, an average percentage was taken to provide a *decision-making score*, which was calculated for pre-CISP, the first half, and second half, of the CISP.

After responding initially, participants had 30-s where they were asked to evaluate their response during active recovery. Specifically, participants were asked to provide a reason for their choice, drawing on anything which helped influence their decision such as space, player movement and what could happen next. This method was informed by similar “think-aloud” methods employed in previous research where verbalisations are collected as athletes engage in exercise to investigate cognition [e.g., 27,42]. All decision-making clips were projected onto a television screen positioned outside the viewing window of the environmental chamber, directly in front of the participant. An omnidirectional boundary microphone (Samson CM11B, Samson Technologies, New York, US) was attached to the cycle ergometer to collect verbal responses and was connected via a small port to a laptop located outside of the chamber.

Panopto software (Panopto 2020, Pittsburgh, US) was used to sync the video footage which was played from the laptop outside of the chamber, to the verbalised reports. Each recording was then downloaded as an MP4 file and transcribed ready for analysis in NVivo (QSR International 2020, Massachusetts, US). Inductive content analysis was used to analyse the recordings obtained from the participants [42,43], allowing themes to emerge from the raw data as no similar research had been undertaken in soccer players to base pre-existing themes on. Firstly, verbalisations were coded openly where inductive themes continued to be generated from the raw data throughout the content analysis process. These codes were then categorised into themes and underlying sub-themes (See Table 2). The number of verbalisations for each theme and sub-theme was identified for each participant across the temperature conditions in NVivo and then recorded in Excel (Version 2008, Microsoft).

#### Challenge and threat states

To assess challenge and threat states, demand and resource evaluations were measured to determine a cognitive appraisal ratio [44] pre-CISP and every 3^rd^, 9^th^, 15^th^ and 21^st^ sprint in each half. At rest, participants were asked “How demanding do you expect the protocol to be?” and “How able are you to cope with the demands of the protocol?” to measure demand evaluations and resource evaluations, respectively. The tense of these questions was then adapted for during the CISP (e.g., “How demanding are you finding the protocol?”). These two items were ranked using a 6-point Likert scale anchored from 1 (*not at all*) to 6 (*extremely*). A ratio was calculated by dividing demands by resources, where a value greater than 1 indicated a threat state, and less than 1 indicated a challenge state [44].

#### Affect

Affective valence and activation were measured pre-CISP and every 3^rd^, 9^th^, 15^th^ and 21^st^ sprint of each half of the CISP using the Feeling Scale [45] anchored from -5 (*very bad*) through 0 (*neutral*) to 5 (*very good*). Unick et al. [46] also found excellent agreement during exercise within individuals across three sessions for the Feeling Scale (ICC = 0.83).

#### Plasma metanephrines

For measurement of plasma metanephrines, 3ml blood samples were collected in *ethylenediamine tetraacetic acid* (EDTA) tubes from an indwelling venous catheter located in the arm at pre-CISP, half-time, and immediately post-CISP. These were centrifuged immediately (ALC; PK120R) and plasma separated for analysis of plasma metadrenaline (MET) and normetanephrine (NMET), measured as the more stable metabolites of plasma epinephrine and norepinephrine, respectively [47]. These samples were frozen at -80°c until analysis by liquid chromatography–tandem mass spectrometry in the Integrated Laboratory Medicine Department, Freeman Hospital.

### Additional measures

#### Core temperature (T_C_)

T_C_ was measured using a temperature sensor telemetry system in the form of a small ingestible pill. Participants ingested the thermometer pill ~6 hours prior to exercise [48]. T_C_ was first recorded pre-trial following 15-min of seated rest to allow resting values to stabilise. T_C_ was then recorded 1-min into each 2-min block, allowing a period for T_C_ to stabilise following the 5-s sprint [39], and was also recorded this regularly for participant safety reasons. The T_C_ recordings were averaged across 5-time points for each half of the CISP (i.e., every 10-min and the last 6-min of each 46-min half) for analysis.

#### Heart rate

After sitting for 15-min to allow resting values to stabilise, participants HR (beats per min) was recorded using a polar HR monitor (FT1, Polar, Finland). HR was then recorded immediately after every sprint throughout the CISP, then averaged across 5-time points for each half of the CISP (i.e., every 10-min and the last-6-min of each 46-min half) for analysis.

#### Fluid loss and hydration status

After being towelled off, participants were asked to enter a secure room, undress, and step onto the Tanita scales to measure nude body mass immediately pre- and post-each CISP, to determine fluid loss from the trial [49]. Urine osmolality was also measured immediately prior to nude body mass pre-CISP, where participants were asked to provide a urine sample which was subsequently analysed on an osmometer (Osmomat 030, Genotec, Germany). Participants were then asked to provide a urine sample post-CISP, this time following the nude body mass procedure as per previous research [50], due to needing to account for fluid intake when calculating fluid lost during the trial.

#### Thermal sensation

Thermal sensation (TSS) was recorded every 3^rd^, 9^th^, 15^th^ and 21^st^ sprint in each half of the CISP using Toner et al.’s [51] 0 (*unbearably cold*) to 8 (*unbearably hot*) Likert scale. This thermal sensation scale has been shown to be a valid measure of perceived heat stress, where correlations with large effect sizes (*r* = .72) have been shown between thermal sensation ratings and rectal temperature [52].

#### Rating of perceived exertion

Ratings of perceived exertion (RPE) were taken every 3^rd^, 9^th^, 15^th^ and 21^st^ sprint in each half of the CISP, using the 6–20 Borg scale [53]. Previous research assessing the reliability of the Borg Scale has found excellent agreement during exercise (ICC = 0.77) within individuals across three sessions [46].

### Data analysis

Statistical analysis was completed using IBM SPSS statistics 24.0 (IBM Corporation). After data screening and checking for normality of the data, a series of two-way (experimental condition by time) repeated measures ANOVAs were performed. Firstly, to analyse differences in indicators of physiological (e.g., core temperature, heart rate, fluid loss) and perceived psychological (e.g., thermal sensation, RPE) strain. This was to check whether participants were under greater strain in the hot compared to the temperate condition. Then, a series of two-way repeated measures ANOVAs were performed to address our hypotheses. Specifically, to examine differences in physical (i.e., PPO) and cognitive (i.e., decision-making) performance, perceived challenge and threat appraisals, affective valence, and plasma metanephrines. Homogeneity of variance was assessed using Mauchly’s test of Sphericity. Where homogeneity of variance was unable to be assumed, Greenhouse-Geisser corrections were applied. Where significant results were identified, Bonferroni pairwise comparisons post-hoc tests were performed. Two-tailed statistical significance was accepted at *p* ≤ 0.05. All data were reported as means and standard deviation (*SD*). For visual clarity, Figure error bars represent standard error mean (SEM).

Partial eta-squared (η_p_^2^) and generalised eta-squared (η^2^_G_) are reported as the effect size for ANOVAs. Due to Cohen’s [54] original partial eta-squared benchmarks of .01, .06 and .14 for small, medium, and large effects, respectively, not being suitable for interpreting partial eta-squared for multi-factorial within-subjects designs [55], these benchmarks were compared to generalized eta-squared which address considerations for which these benchmarks were based [55]. Additionally, Cohen’s *d* was reported as the effect size for pairwise comparisons. In accordance with Cohen [56], *d* values of 0.2, 0.5 and 0.8 indicated small, moderate, and large effects for pairwise comparisons, respectively.

## Results

### Preliminary analyses

#### Core temperature

**Fig 2:**
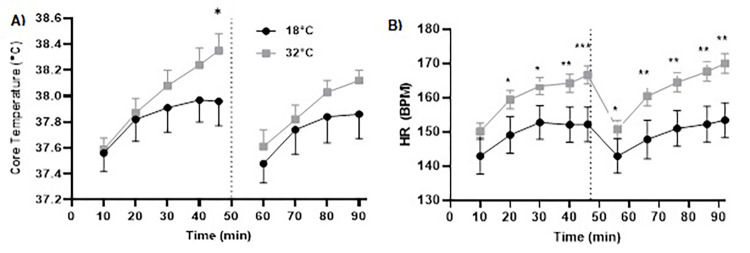
Core Temperature (Panel A) and HR (BPM) (Panel B) throughout the 92-min CISP between conditions. *Note*: * *p ≤* .05, ** *p ≤* .01, *** *p* ≤ .001.

A condition (2) × time (10; 10-min intervals and the last 6-min of each half) ANOVA for T_C_ revealed a main effect for time (*F*_9,72_ = 16.142, *p* ≤ .001, η_p_^2^ = .669, η^2^_*G*_ = .188) and an interaction effect (*F*_9,72_ = 2.073, *p* = .043, η_p_^2^ = .206, η^2^_*G*_ = .020), but no effect for condition (*F*_1,8_ = 1.531, *p* = .251, η_p_^2^ = .161, η^2^_*G*_ = .050). Core temperature was higher in hot compared to the temperate condition in the final 6-min of the first half (i.e., 40^th^-46^th^-min) (*p* = .046, *d* = 0.82), but was not different between conditions at any other time-point (*ps* ≥ .107, *d*s ≥ 0.10 to 0.84) (see Fig 2A).

#### Heart rate

There were condition (*F*_1,8_ = 13.581, *p* = .006, η_p_^2^ = .629, η^2^_*G*_ = .209), time (*F*_9,72_ = 33.384, *p* ≤ .001, η_p_^2^ = .807, η^2^_*G*_ = .155), and condition (2) × time interaction (10; 10-min intervals and the last 6-min of each half) effects found for HR (*F*_9,72_ = 3.375, *p* = .002, η_p_^2^ = .297, η^2^_*G*_ = .016). Heart rate was significantly higher in the hot compared to temperate condition at all time-points (*ps* ≤ .042, *d*s = 0.66 to 1.34), apart from in the first 10-min of the first half of the CISP (*p* = .154, *d* = 0.58) (See Fig 2B).

#### Fluid loss (ml), urine osmolality (mOsm) and fluid intake (ml)

There was a significant condition (*F*_1,8_ = 47.901, *p* ≤ .001, η_p_^2^ = .857, η^2^_*G*_ ≤ .001) effect, whereby fluid loss was significantly higher in the hot (*M*^*diff*^ = -1.60 ± 0.30 kg) compared to the temperate (*M*^*diff*^ = -0.80 ± 0.40 kg) condition. For urine osmolality, no condition (*F*_1,8_ = 1.224, *p* = .301, η_p_^2^ = .133, η^2^_*G*_ = .036), time (*F*_1,8_ = 0.709, *p* = .424, η_p_^2^ = .081, η^2^_*G*_ = .012) nor condition × time interaction (*F*_1,8_ = 3.068, *p* = .118, η_p_^2^ = .277, η^2^_*G*_ = .049) effect was found. Additionally, there was a main condition effect for fluid intake whereby participants consumed more fluid (water) in the hot (1067 ± 440 ml) compared to the temperate (728 ± 168 ml) condition (*F*_1,8_ = 7.525, *p* = .025, η_p_^2^ = .485, η^2^_*G*_ = .218).

#### Rate of perceived exertion

**Fig 3:**
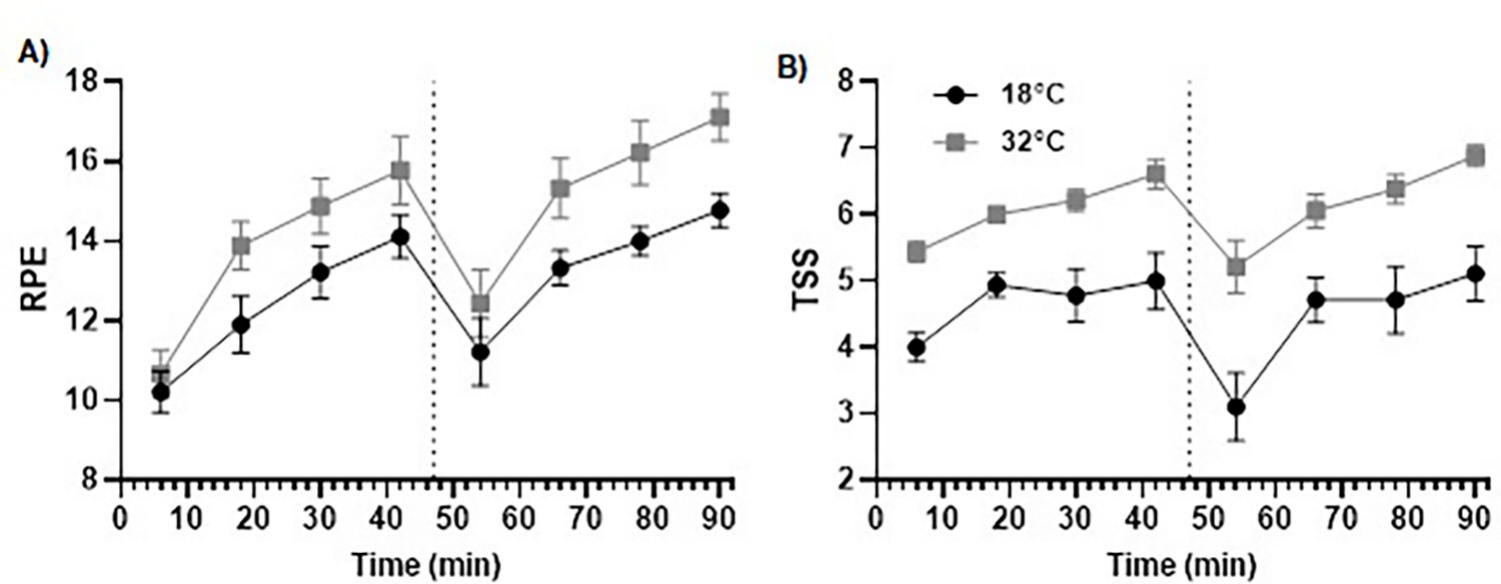
RPE (Panel A) and TSS (Panel B) during the 92-min CISP between conditions.

A 2 (condition) by 8 (time; 6^th^-min then 12-min intervals throughout the CISP) ANOVA identified effects for condition (*F*_1,8_ = 10.914, *p* = .011, η_p_^2^ = .577, η^2^_*G*_ = .171) and time (*F*_7,56_ = 20.451, *p* ≤ .001, η_p_^2^ = .719, η^2^_*G*_ = .461), but no interaction effect (*F*_7,56_ = 1.155, *p* = .343, η_p_^2^ = .126, η^2^_*G*_ = .023). RPE was significantly higher in the hot (14.54 ± 1.65) compared to the temperate (12.85 ± 1.08, *p* = .011, *d* = 1.21) condition. In terms of time, Bonferroni pairwise comparisons identified that RPE was significantly higher toward the end of the first half (i.e., 42^nd^-min) (14.94 ± 1.82, *p* ≤ .001, *d* = 2.66), midway through the second half (i.e., 78^th^ min) (15.11 ± 1.17, *p* ≤ .001, *d* = 3.40) and at the end of the second half of the CISP (i.e., 90^th^ min) (15.94 ± 1.01, *p* ≤ .001, *d* = 4.20), compared to the start of the first half of the CISP (i.e., the 6^th^ min) (10.44 ± 1.55). RPE was also significantly lower at the start of the second half (i.e., 54^th^ min) (11.83 ± 2.45) compared to the end of the second half (i.e., 90^th^ min) (15.94 ± 1.01, *p* = .024, *d* = 2.19) (see Fig 3A).

#### Thermal sensation

A 2 (condition) by 8 (time; 6^th^-min then every 12-min intervals throughout the CISP) ANOVA found significant main effects for condition (*F*_1,8_ = 51.213, *p* ≤ .001, η_p_^2^ = .865, η^2^_*G*_ = .426) and time *(F*_7,56_ = 9.101, *p* ≤ .001, η_p_^2^ = .532, η^2^_*G*_ = .277), but no condition × time interaction effect (*F*_7,56_ = 1.403, *p* = .222, η_p_^2^ = .149, η^2^_*G*_ = .026) for TSS. Specifically, the effect of condition revealed that TSS was significantly higher in the hot (6.10 ± 0.42) compared to temperate (4.55 ± 0.87, *p* ≤ .001, *d* = 2.11) condition. For the time effect, Bonferroni pairwise comparisons identified higher TSS in the 18^th^ (5.47 ± 0.45, *p* = .030, *d* = 1.63) and 90^th^-min (6.00 ± 0.75, *p* = .037, *d* = 2.05) compared to the 6^th^-min (4.72 ± 0.47) (see Fig 3B).

#### Summary of preliminary analyses

Overall, the findings from the preliminary analyses revealed that in the hot condition, participants showed greater cardiovascular strain (increased heart rate), higher fluid loss, felt hotter (i.e., higher TSS), perceived the exercise more demanding or exerting (higher RPE). Moreover, although no main condition effect was found for core temperature, core temperature was still higher at the end of each half of the CISP in the hot condition compared to the temperate condition. Therefore, these results are suggestive that participants were under greater physiological and perceived psychological strain during exercise in the hot condition.

### Performance measures

#### Peak power output

**Fig 4:**
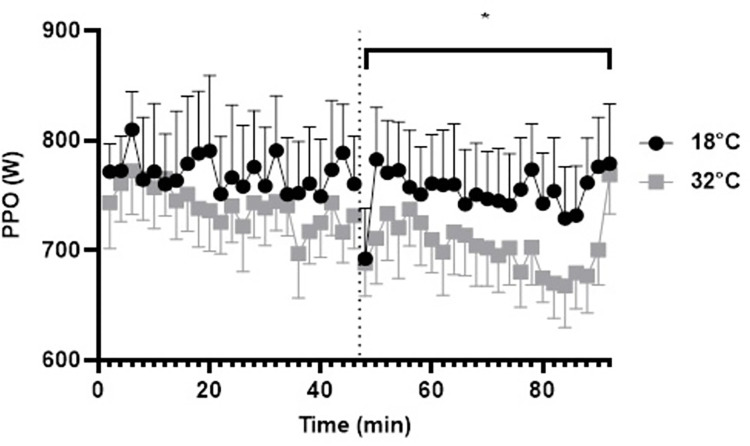
Peak power output across all 5-s sprints during the 92-min CISP; the dashed line represents half-time. *Note*: * *p ≤*.05 for condition × time interaction.

A 2 (condition) by 2 (time; first half, second half) ANOVA revealed no effect for time (*F*_1,8_ = 4.976, *p* = .056, η_p_^2^ = .383, η^2^_G_ = .010) or condition (*F*_1,8_ = 2.259, *p* = .171, η_p_^2^ = .220, η^2^_G_ = .029). However, a significant condition × time interaction (*F*_1,8_ = 6.659, *p* = .033, η_p_^2^ = .454, η^2^_G_ = .010) was found (See Fig 4). Bonferroni pairwise comparisons identified that PPO was significantly lower in the second half of the CISP in 32˚C (705 ± 93 W) compared to the first half (755 ± 136 W; *p* = .016, *d* = 0.44), but was not significantly different in the second half (739.12 ± 92.73 W) compared to the first half (768 ± 155 W; *p* = .313, *d* = 0.22) of the CISP in 18˚C (see Fig 2). Bonferroni comparisons identified no significant differences between the hot and temperate condition in the first half (*M*^diff^ = -28 ± 27 W, *p* = .328, *d* = 0.08) or second half of the CISP (*M*^diff^ = -51 ± 26 W, *p* = .087, *d* = 0.37).

#### Decision-making score (%)

**Fig 5:**
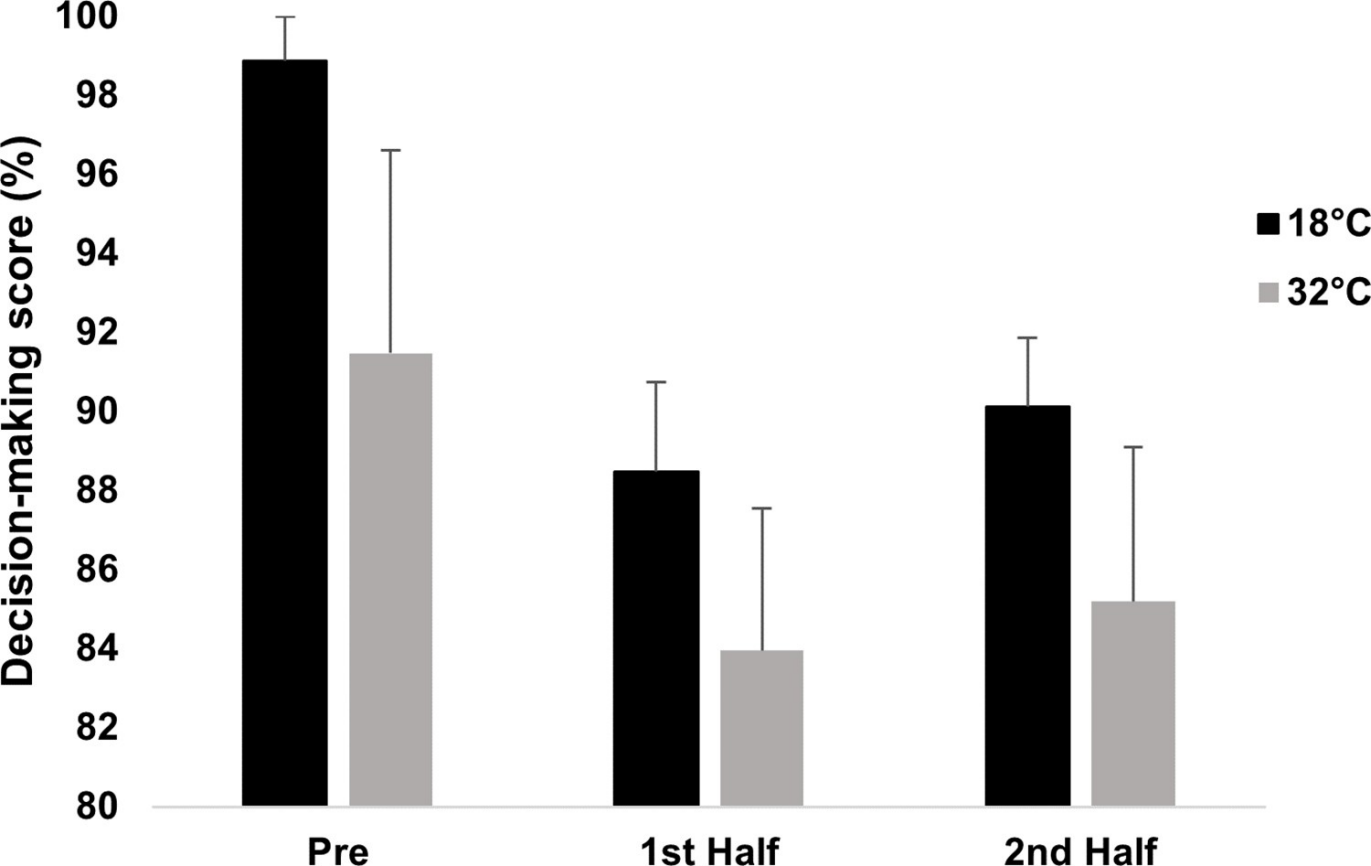
Decision-making score (%) in 18˚C and 32˚C across pre-CISP, first and second half of the CISP. *Note*: * *p ≤* .05 for main effect of condition.

A 2 (condition) by 3 (time; pre-CISP; first half and second half) ANOVA revealed significant main effects for condition (*F*_1,8_ = 6.930, *p* = .030, η_p_^2^ = .464, η^2^_G_ = .085) and time (*F*_2,16_ = 3.859, *p* = .043, η_p_^2^ = .325, η^2^_G_ = .153), but no condition × time interaction effect (*F*_2,16_ = 0.156, *p* = .857, η_p_^2^ = .019, η^2^_G_ = .001) (See Fig 5). In terms of the condition effect, decision-making scores were lower in 32˚C (86.87 ± 7.95%) than in 18˚C (92.49 ± 3.13%, *p* = .03, *d* = 0.93) (see Fig 5). In terms of the time effect, although Bonferroni comparisons revealed no significant differences, means reflected lower decision-making scores in both first (86.22 ± 6.42%, *p* = .179, *d* = 1.13) and second (87.66 ± 7.80%, *p* = .187, *d* = 0.88) halves of the CISP, compared to pre-CISP (95.17 ± 9.20%).

### Decision-making evaluation

#### Themes and initial descriptive analysis

From the inductive content analysis, 4 themes and 14 sub-themes were extracted. The themes reflected a) ‘game intelligence’, used for verbalisations which recognised critical information as it occurred such as player movement, goal-scoring opportunities, tactical awareness, and/or showed deeper planning as to what could or should occur following the initial decision; b) ‘risk management’, used for verbalisations which identified potential threat or discussed risk in relation to the available options; c) ‘difficulty’, used to categorise verbalisations which referenced how difficult the decision made would be to execute, or when participants referred to the difficulty, or showed hesitation toward the decision-making process; and finally d) ‘lack of evaluation’, used to reflect responses where the decision made lacked rationale and/or where participants simply re-described the action they would take (see Table 1). Across conditions, verbalisations were made in relation to ‘game intelligence’ most frequently (~57%), followed by ‘risk management’ (~18%), ‘difficulty’ (~8%) and ‘lack of evaluation’ (~3%). The remaining verbalisations consisted primarily of making the initial decision (~14%).

**Table 1:** Content analysis for participant response evaluations and total number of verbalisations.

Themes	Sub-Themes	Description	Examples of Raw Data
**Difficulty (61)**	**Difficult Execution (16)**	Reference to the decision being difficult to execute	“You can’t get an easy shot off” “…the angles too tight to shoot” “…difficult pass but if you made that pass, you’d have a good chance of scoring”
	**Easy Execution (30)**	Reference to the decision being an easy one to execute	“…it’s an easy tap in” “…an easy shot to take” “…his body shape shows it’s the easiest pass”
	**Uncertainty (16)**	Uncertainty is shown as to the decision itself or whether the decision made would be possible to execute successfully	“What is that clip, what would you do?” “If he can, play it to blue” “If he gets the ball, he might be able to have a cross”
**Risk Management (121)**	**Perceived Threat (14)**	Immediate reference to threat / danger for the player on the ball	“He’s surrounded by players so it’s risky trying to play a through-ball” “…because I could be in danger of losing it in the area” “The player was tracking him down so it would have been risky trying to play it to the other players”
	**Risk Assessment (34)**	Reference to risk associated with the available options	“You can’t play it back to blue because he’s already got the keeper on him and yellow is surrounded by players” “Shoot as I’m more than likely to lose it if I pass anywhere else” “If he tried to pass it, he’d probably lose the opportunity”
	**Restricted Options (32)**	Reference to there being limited options available	“There’s not much on at the moment” “I’d keep driving into space because the options of passing aren’t that good” “All the other players around him are marked so that’s the only real option he’s got”
	**‘Safe’ Option (23)**	Reference to retaining possession, not losing possession, or going backwards to reset play	“…back to keeper, he’s under a lot of pressure so start again” “…just to keep hold of the ball” “…go back, I’d say the other options were quite difficult”
	**Available Time (11)**	Reference to the amount of time a player has or controlling time	“Just take your time there’s no need to rush it” “There’s people on him and no time” “…but he’s going to be pressed quickly so he needs to release the ball quickly”
**Game Intelligence (474)**	**Future Planning (60)**	Reference to what actions might/should occur following the initial decision	“…for him to then set it back to me so I can break the line of defence”“I’d play a 1–2, to get it back through on goal or receive it back and slot it along the box for someone to run onto” “…there’s a lot of space to run into where he can get a cross off for other players running in”
	**Chance of Scoring (173)**	Reference to creating or having a goal-scoring opportunity	“There’s a clear opportunity on goal, so shoot” “I’d have a shot because I’m pretty much one on one with the keeper” “…the balls set on the half volley so he should shoot”
	**Space/Positioning (167)**	Reference to areas of space on the pitch and player positioning	“He’s got heaps of space to run into” “He’s in the most space with fewer people around him” “The players in between the midfield and defence so he’s in quite a good packet of space”
	**Tactical Awareness (50)**	Reference to tactical play e.g., identifying the oppositions high line, acknowledging offside runs	“The back line is too deep” “He was in and around the box so might be able to get a penalty” “He’s onside, can receive it on the back foot and he’s through on goal”
	**Player Movement (21)**	Reference to specific player movement to inform the decision	“He’s on the half-turn, he’s already making the run” “He’s asking for the ball” “He’s running in toward the box, he’ll be able to get a shot at goal”
**Lack of Evaluation (20)**	**Lack of Evaluation (20)**	Little reference made evaluating or reasoning the initial response	“You might as well shoot” “Maintain possession down the line” “He’s got nothing else to do really”

*Notes*: Meaning units (i.e., number of times the theme is mentioned) for all verbalisations across conditions are reported in brackets for each theme for 8 participants.

#### Statistical analyses for themes between conditions

Square root transformation was performed to allow difference testing for theme frequency across conditions and time for such count data in SPSS [57]. Inferential statistics in-text are reported from the transformed data, where data presented in Tables 1 and 2 is reported as non-transformed data. A series of two-way (2 condition × 2 time; first half and second half) repeated measures ANOVAs were run to identify whether there were differences in participants’ overall verbalisations during the first and second half of the CISP, across conditions (see Table 2). There were no main effects for condition (*F*_1,7_ = 0.476, *p* = .513, n_p_^2^ = .064, η^2^_G_ = .009) or time (*F*_1,7_ = 1.060, *p* = .337, n_p_^2^ = .132, η^2^_G_ = .035), nor a condition × time interaction effect (*F*_1,7_ = 0.937, *p* = .365, n_p_^2^ = .118, η^2^_G_ = .014) for the number of themes extracted. Additionally, no condition (*F*_1,7_ = 0.268, *p* = .620, n_p_^2^ = .037, η^2^_G_ = .011), time (*F*_1,7_ = 2.038, *p* = .197, n_p_^2^ = .225, η^2^_G_ = .019) or condition × time interaction (*F*_1,7_ = 0.708, *p* = .428, n_p_^2^ = .092, η^2^_G_ = .003) effects were identified for total number of verbalisations (see Table 1).

**Table 2:** Verbalised themes (non-transformed data) during exercise between conditions.

	Condition		Time		Condition × Time			
Theme	18°C	32°C	1^st^ Half	2^nd^ Half	18°C 1^st^ Half	18°C 2^nd^ Half C2	32°C 1^st^ Half	32°C 2^nd^ Half
**Total Themes**	7.20 ± 1.72	7.44 ± 1.90	7.44 ± 1.55	7.20 ± 2.04	7.38 ± 1.51	7.00 ± 2.00	7.50 ± 1.69	7.40 ± 2.20
**Total Verbalisations**	17.50 ± 4.30	18.63 ± 5.77	18.81 ± 6.04	17.31 ± 3.81	18.00 ± 5.45	17.00 ± 2.93	19.63 ± 6.84	17.63 ± 4.72
**Difficulty**	2.06 ± 1.46	1.88 ± 1.50	1.94 ± 1.81	1.44 ± 1.75	1.88 ± 1.46	1.13 ± 1.46	2.00 ± 2.20	1.75 ± 2.05
Difficult Execution	0.56 ± 0.81	0.38 ± 0.62	0.63 ± 0.81	0.31 ± 0.60	0.88 ± 0.99	0.25 ± 0.46	0.38 ± 0.52	0.38 ± 0.74
Easy Execution	0.96 ± 1.26	0.63 ± 0.88	1.06 ± 0.44	0.44 ± 0.73	1.25 ± 1.58	0.50 ± 0.76	0.88 ± 1.13	0.38 ± 0.74
Uncertainty	0.25 ± 0.60	0.75 ± 1.57	0.31 ± 1.01	0.69 ± 1.40	0.13 ± 0.35	0.38 ± 0.74	0.50 ± 1.41	1.00 ± 1.77
**Risk Management**	3.13 ± 2.94	3.63 ± 3.88	3.69 ± 3.59	3.06 ± 3.28	3.50 ± 2.40	2.75 ± 2.54	3.88 ± 4.67	3.76 ± 3.20
Perceived Threat	0.44 ± 0.51	0.44 ± 0.51	0.56 ± 0.51	0.31 ± 0.48	0.63 ± 0.52	0.25 ± 0.46	0.50 ± 0.53	0.38 ± 0.52
Risk Assessment	0.81 ± 1.22	0.94 ± 1.34	0.88 ± 1.41	0.88 ± 1.15	0.75 ± 1.16	0.88 ± 1.36	1.00 ± 1.69	0.88 ± 0.99
Restricted Options	0.69 ± 1.30	1.06 ± 1.29	0.56 ± 1.03	1.89 ± 1.47	0.25 ± 0.46	1.13 ± 1.73	0.88 ± 1.36	1.25 ± 1.28
Safe Option	0.44 ± 0.89	0.81 ± 1.22	0.88 ± 1.36	0.38 ± 0.62	0.63 ± 1.19	0.25 ± 0.46	1.13 ± 1.55	0.50 ± 0.76
Available Time	0.38 ± 0.50	0.31 ± 0.48	0.38 ± 0.50	0.31 ± 0.48	0.50 ± 0.53	0.25 ± 0.46	0.25 ± 0.46	0.38 ± 0.52
**Game Intelligence**	12.44 ± 2.87	12.25 ± 4.28	12.81 ± 4.16	11.88 ± 2.96	12.40 ± 3.82	12.50 ± 1.77	13.25 ± 4.71	11.25 ± 3.85
Future Planning	**2.13 ± 1.15**	0.88 ± 1.31*	1.44 ± 1.46	1.56 ± 1.32	1.88 ± 1.13	2.40 ± 1.19	1.00 ± 1.13	0.75 ± 0.89
Chance of Scoring	4.50 ± 1.16	4.69 ± 1.78	4.69 ± 0.95	4.50 ± 1.90	4.50 ± 1.07	4.50 ± 1.31	4.88 ± 0.83	4.50 ± 1.31
Space & Positioning	4.44 ± 1.79	4.38 ± 1.63	4.25 ± 1.57	4.56 ± 1.83	4.50 ± 1.86	4.25 ± 1.49	4.00 ± 1.31	4.88 ± 2.17
Tactical Awareness	1.13 ± 1.03	1.50 ± 1.63	**1.81 ± 1.56**	0.81 ± 0.91*	1.38 ± 1.06	0.88 ± 0.99	2.25 ± 1.91	0.75 ± 0.89
Player Movement	0.31 ± 0.48	0.75 ± 1.00	0.63 ± 0.96	0.44 ± 0.63	0.13 ± 0.35	0.50 ± 0.53	**1.13 ± 1.13**	0.38 ± 0.75*
**Lack of Evaluation**	0.38 ± 0.81	0.88 ± 0.81	**0.31 ± 0.60**	0.94 ± 0.93*	0.13 ± 0.35	0.63 ± 1.06	0.50 ± 0.76	1.25 ± 0.71

Note: * = significant difference *p ≤* .*05*, all significant differences are highlighted in bold.

#### Difficulty

For ‘difficulty’, no main condition (*F*_1,7_ = 0.010, *p* = .921, n_p_^2^ ≤ .001), time (*F*_1,7_ = 4.640, *p* = .068, n_p_^2^ = .399, η^2^_G_ = .042), nor condition × time interaction (*F*_1,7_ = 1.712, *p* = .232, n_p_^2^ = .196, η^2^_G_ = .025) effects were observed. Within the sub-themes underpinning ‘difficulty’, there were no condition (*p*s > .375 to .679, n_p_^2^ = .026 to .113, η^2^_G_ = .010 to .027), time (*p*s > .078 to .256, n_p_^2^ = .247 to .378, η^2^_G_ = .041 to .063), nor condition × time interaction (*p*s > .076 to ≥.999, n_p_^2^ = ≤.001 to .381, η^2^_G_ = ≤.001 to .038) effects for ‘difficult execution’, ‘easy execution’ and ‘uncertainty’.

#### Risk management

For ‘risk management’, no condition (*F*_1,7_ = 0.045, *p* = .839, n_p_^2^ = .006, η^2^_G_ = .001), time (F_1,7_ = 1.058, *p* = .338, n_p_^2^ = .131, η^2^_G_ = .025) or condition × time interaction (*F*_1,7_ = 0.355, *p* = .570, n_p_^2^ = .048, η^2^_G_ = .005) effects were noted. For the sub-themes underpinning ‘risk management’, no condition (*p*s = .169 to 1.000, n_p_^2^ = ≤.001 to .252, η^2^_G_ ≤ .001 to .038), time (*p*s = .210 to .762, n_p_^2^ = .014 to .214, η^2^_G_ = .001 to .059) or condition × time interaction (*p*’s = .197 to .764, n_p_^2^ = .014 to .225, η^2^_G_ = .001 to .039) effects were found for ‘perceived threat’, ‘restricted options’, ‘risk assessment’ ‘safe options’ or ‘available time’.

#### Game intelligence

For ‘game intelligence’, no condition (*F*_1,7_ = 0.061, *p* = .813, n_p_^2^ = .009, η^2^_G_ = .003), time (*F*_1,7_ = 0.494, *p* = .505, n_p_^2^ = .066, η^2^_G_ = .013) or condition × time interaction (*F*_1,7_ = 1.294, *p* = .293, n_p_^2^ = .156, η^2^_G_ = .026) effects were observed. For sub-themes underpinning ‘game intelligence’, a main effect for condition (*F*_1,7_ = 7.423, *p* = .030, n_p_^2^ = .515, η^2^_G_ = .332) was found for ‘future planning’, but there was no time (*F*_1,7_ = 0.160, *p* = .701, n_p_^2^ = .022, η^2^_G_ = .003) nor condition × time interaction (*F*_1,7_ = 0.584, *p* = .470, n_p_^2^ = .077, η^2^_G_ = .011) effects. Specifically, a lower number of “future planning” verbalisations were made in the hot (0.99 ± 0.42) compared to the temperate condition (1.05 ± 0.11, d = 0.20). For ‘chance of scoring’ and ‘space and positioning’, no condition (*ps* = .882 to .965, n_p_^2^ ≤ .001 to .003, η^2^_G_ ≤ .001 to .002), time (*p*s = .545 to .590, n_p_^2^ = .590 to .055, η^2^_G_ = .006 to .014), or condition × time interaction (*p*s = .533 to .659, n_p_^2^ = .029 to .058, η^2^_G_ = .011 to .021) effects were found.

For ‘tactical awareness’, no condition (*F*_1,7_ = 0.201, *p* = .668, n_p_^2^ = .028, η^2^_G_ = .006) or condition × time interaction (*F*_1,7_ = 0.802, *p* = .400, n_p_^2^ = .103, η^2^_G_ = .013) effect was found. A main effect for time (*F*_1,7_ = 5.724, *p* = .048, n_p_^2^ = .450, η^2^_G_ = .117) was identified whereby significantly less ‘tactical awareness’ statements were made in the second half (0.63 ± 0.62) compared to the first half (1.13 ± 0.59, *d* = 0.83). For ‘player movement’, no main condition (*F*_1,7_ = 1.127, *p* = .324, n_p_^2^ = .139, η^2^_G_ = .053) or time (*F*_1,7_ = 0.082, *p* = .782, n_p_^2^ = .012, η^2^_G_ = .005) effects were identified. However, a condition × time interaction effect (*F*_1,7_ = 12.021, *p* = .010, n_p_^2^ = .632, η^2^_G_ = .154) was observed whereby ‘player movement’ verbalisations were lower in the first half in the hot compared to the temperate condition (*M*^diff^ = -0.70 ± 0.68, *p* = .024, *d* = 0.80), though they were not different in the second half (*M*^diff^ = -0.20 ± 0.82, *p* = .515, *d* = 0.82).

#### Lack of evaluation

In terms of ‘lack of evaluation’, a main effect for time was found (*F*_1,7_ = 8.340, *p* = .023, n_p_^2^ = .544, η^2^_G_ = .179), where more responses lacked evaluation in the second half (0.75 ± 0.48) compared to the first half (0.28 ± 0.40, *p* = .023, *d* = 1.06) of the CISP. There was no effect for condition (*F*_1,7_ = 5.068, *p* = .059, n_p_^2^ = .420, η^2^_G_ = .155), nor condition × time interaction (*F*_1,7_ = 2.232, *p* = .179, n_p_^2^ = .242, η^2^_G_ = .016).

### Effect of heat on appraisal, affective valence and metadrenalines

#### Challenge and threat states

**Fig 6:**
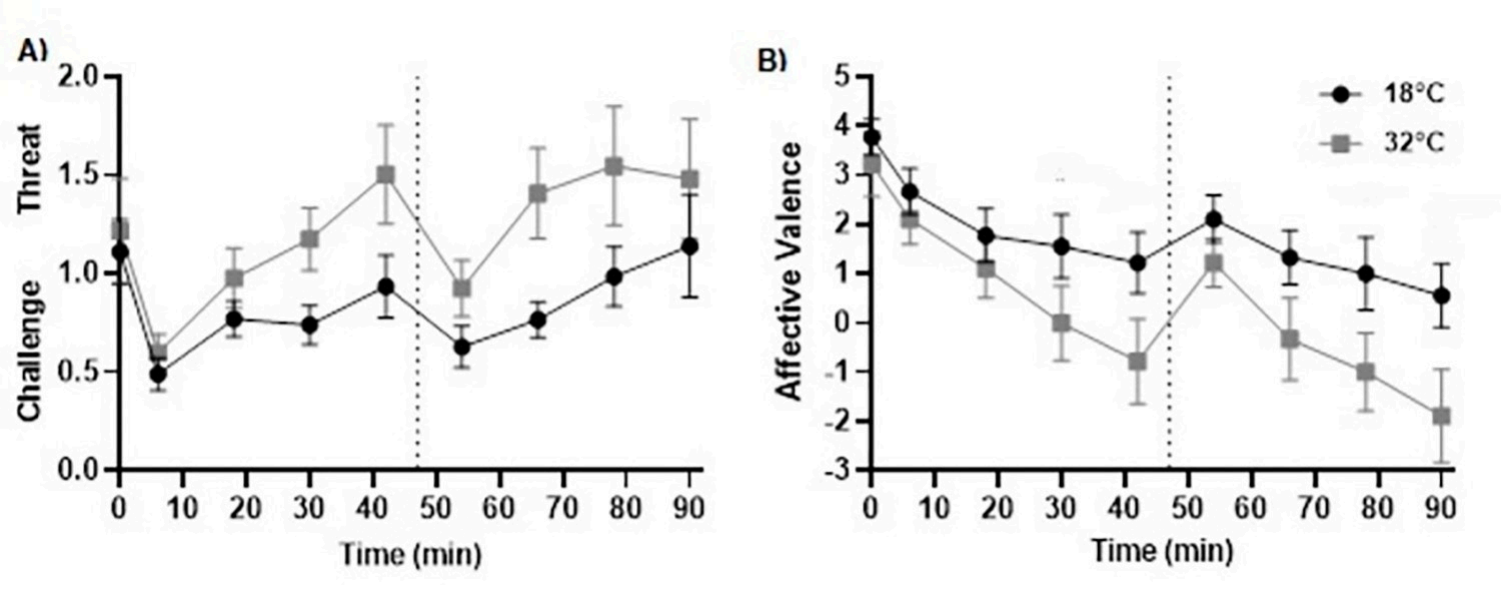
Challenge and threat state (Panel A) and affective valence (Panel B) across the 92-min CISP between conditions.

A 2 (condition) by 9 (time; pre-CISP, 6^th^-min then 12-min intervals throughout the CISP) ANOVA revealed significant main effects for condition (*F*_1,8_ = 13.069, *p* = .007, η_p_^2^ = .620, η^2^_*G*_ = .104) and time (*F*_8,64_ = 6.769, *p* ≤ .001, η_p_^2^ = .458, η^2^_*G*_ = .169), but no interaction effect was identified (*F*_8,64_ = 1.050, *p* = .409, η_p_^2^ = .116, η^2^_*G*_ = .031). Post-hoc analysis revealed a higher threat state in the hot condition (1.20 ± 0.50) compared to a higher challenge state in temperate condition (0.84 ± 0.29, *p =* .007, *d* = 0.88). For the effect of time, no significant differences were observed between time points (*p*s > .070, *d*s < 1.73), apart from between the 6^th^ (.55 + .21) and 66^th^ minute (1.09 ± .15, *p* = .05, *d* = 1.54) (see Fig 6A).

#### Affective valence

For affective valence, a 2 (condition) by 9 (time; pre-CISP, 6^th^-min, and then 12-min intervals throughout the CISP) ANOVA found significant effects for condition (*F*_1,8_ = 12.417, *p* = .008, η_p_^2^ = .608, η^2^_*G*_ = .119) and time (*F*_8,64_ = 12.159, *p* ≤ .001, η_p_^2^ = .603, η^2^_*G*_ = .299), but no condition × time interaction effect (*F*_8,64_ = 1.991, *p* = .067, η_p_^2^ = .196, η^2^_*G*_ = .032). Post-hoc analysis revealed that affective valence was significantly lower in 32˚C (.41 ± 1.59) in comparison to 18˚C (1.78 ± 1.44, *p* = .008, *d* = .90). Bonferroni pairwise comparisons also identified lower affective valence at the start of the second half (i.e., 50^th^ min) (1.67 ± 1.35, *p* = .030, *d* = 1.28), and in the 66^th^ min (.50 ± 1.80, *p* = .046, *d* = 1.81), and 78^th^ min (0.00 ± 2.01, *p* = .039, *d* = 1.97), compared to rest (3.50 ± 1.50). It was also significantly lower toward the end of the second half in the 66^th^ (.50 ± 1.80, *p* = .045, *d* = 1.19), 78^th^ (.00 ± 2.01, *p* = .025, *d* = 1.40) and 90^th^ min (-.67 ± 2.07, *p* = .042, *d* = 1.75) compared to early in the first half in the 4^th^ min (2.39 ± 1.35) (see Fig 6B).

#### Metanephrines

**Fig 7:**
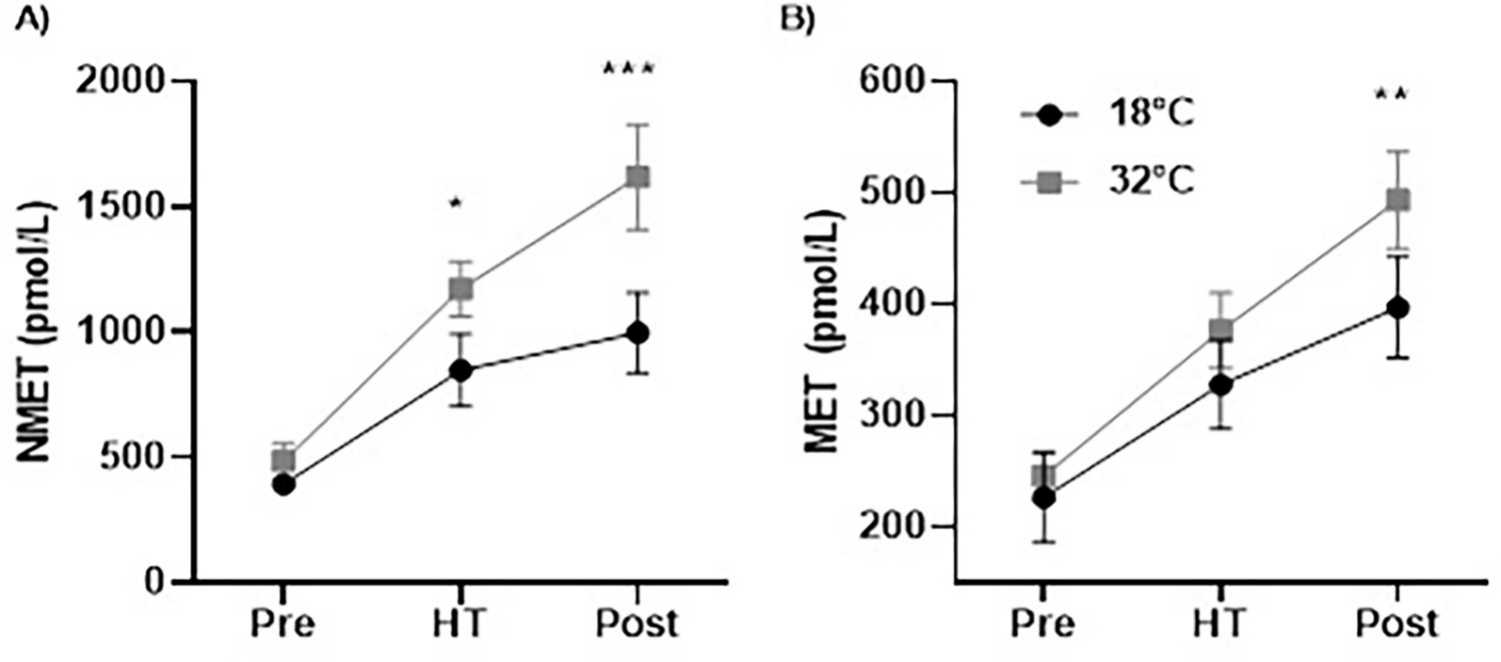
NMET (Panel A) and MET (Panel B) concentrations pre, half-time and post-CISP between conditions. *Note*: * *p* ≤ .05, ** *p* ≤ .01, *** *p* ≤ .001.

Significant condition (*F*_1,7_ = 43.637, *p* ≤ .001, η_p_^2^ = .862, η^2^_*G*_ = .191), time (*F*_1,7_ = 27.187, *p* ≤ .001, η_p_^2^ = .795, η^2^_*G*_ = .506), and condition × time interaction (*F*_1,7_ = 7.278, *p* = .007, η_p_^2^ = .510, η^2^_*G*_ = .085) effects were observed for normetanephrine (NMET) concentration. Specifically, NMET concentration was significantly higher in the hot compared to temperate condition at half-time (*M*^*diff*^ = +325.25 ± 271.50 pmol/L, *p* = .012, *d* = 0.89) and post-CISP (*M*^*diff*^ = +621.63 ± 342.59 pmol/L, *p* ≤ .001, *d* = 1.18), but was not different between the hot and temperate condition pre-CISP (*M*^*diff*^ = +91.88 ± 172.71 pmol/L, *p* = .176, *d* = 0.06) (see Fig 7A).

In terms of metanephrine (MET) concentration, there were significant condition (*F*_1,7_ = 5.954, *p* = .045, η_p_^2^ = .460, η^2^_*G*_ = .067), time (*F*_2,14_ = 37.670, *p* ≤ .001, η_p_^2^ = .843, η^2^_*G*_ = .414), and condition × time interaction (*F*_2,14_ = 6.690, *p* = .009, η_p_^2^ = .489, η^2^_*G*_ = .024) effects. MET concentration was significantly higher in the hot compared to temperate condition post-CISP (*M*^diff^ = +96.63 ± 71.12 pmol/L, *p* = .006, *d* = 0.76), but not pre-CISP (*M*^*diff*^ = +18.88 ± 73.38 pmol/L, *p* = .490, *d* = 0.21) or at half-time (*M*^*diff*^ = +48.50 ± 72.71 pmol/L, *p* = .101, *d* = 0.46) (see Fig 7B).

## Discussion

This study aimed to investigate how soccer-specific decision-making, power output, appraisal, affect, and plasma metanephrines were affected during prolonged intermittent exercise in a hot, compared to a temperate, environment. There was partial support for our first hypothesis (H_1_), whereby soccer-specific decision-making scores (calculated in line with a panel of experts’ decisions) was impaired in 32˚C compared to 18˚C. However, no condition × time effects were observed for decision-making scores which was not aligned to our hypotheses. As expected, intermittent sprint performance was impaired in the second half compared to the first half of the CISP in 32˚C, but did not deteriorate in 18˚C. In support of the second hypothesis (H_2_), ‘future planning’ verbalisations were significantly lower in 32˚C compared to 18˚C, and more responses categorised as ‘lack of evaluation’ were observed in the second half compared to the first half across conditions. This partly reflected lower-level thought processing in the hot condition and following a half of intermittent sprint activity, which partially affirmed our second hypothesis (H_2_). However, we did not find condition × time interactions as originally anticipated. We also found support for our third hypotheses (H_3_), whereby players had a heightened threat state and felt more unpleasant in 32˚C compared to 18˚C. Moreover, MET and NMET concentration were significantly higher following exercise, and these increases were greater in the hot compared to the temperate condition, which reflected a heightened physiological stress response when exercising in hot condition thereby supporting our fourth hypothesis (H_4_). It is worth noting that these findings were similar when analysing data with 8 participants when conditions were fully counterbalanced, and a summary of these findings can be found in the supporting information.

### Heat on cognitive performance

It was anticipated that a more pronounced decline in decision-making score would be observed during the CISP in the hot condition compared to the temperate condition due to additional physiological stress experienced (i.e., higher HR; greater fluid loss; higher T_C_ during latter stages of each half of the CISP) [22,29,31]. Decision-making score was found to be lower throughout all time-points in the hot compared to the temperate condition, but no interaction effect was observed. Resting decision-making was assessed once participants had entered the chamber, and it is possible that passive exposure to hot conditions may have contributed to reducing decision-making performance. Indeed, research has noted that an alteration in subjective state during a short period of passive heat exposure has been shown to impair complex cognitive function [58]. From an occupational perspective, a review by Moda, Filho & Minhas [59] found that increased ambient temperature reduced vigilance levels in outdoor workers, which increased the risk of lapses in safety measures. Previous research has proposed that a T_C_ threshold of ~38.5˚C is where cognitive function becomes impaired [60]. However, these findings suggest that when using more situation, or sport, specific complex decision-making tasks, this threshold may be less sparing where T_C_ did not exceed this threshold in the majority of participants.

A ‘think-aloud’ protocol was also implemented, recording participants retrospective evaluation of each situation to understand what factors were being considered when making the decision, and if this was affected by exercise-induced fatigue or heat stress. Interestingly, thematic analysis revealed that participants verbalised significantly less ‘future planning’ evaluations when in the hot compared to the temperate condition. These findings align with previous research, which found that within 15-min of passive heat exposure (50˚C, 30%rh) complex planning performance is significantly impaired [58]. This decline was attributed to the alliesthesial effect [61], where immediate sensory displeasure and negative affective states induced by a rapid elevation in skin temperature were thought to contribute to the cognitive impairments observed [58]. Heat exposure can be considered an interfering cognitive load, placing further demands on the limited cognitive workspace and thus reducing the availability of neural resources for use in more complex cognitive tasks [58,62].

Verbalisations referencing tactical awareness and execution difficulty were also significantly lower in the second half compared to the first half of the CISP. Additionally, statements which lacked evaluation were higher in the second half compared to the first half, and reference to specific player movement were also significantly reduced in the second half in the hot condition. Support can be drawn from Casanova et al. [27], who found that fatigue induced by prolonged high-intensity intermittent exercise resulted in more impulsive decisions underpinned by lower-level thought processes being observed toward the end of the first half and second half, compared to the beginning of the first half. These findings also align with the transient hypofrontality hypothesis which explains that during physically demanding activity, neural resources are diverted toward regions of the brain responsible for maintaining motor functions, limiting the availability of resources for the pre-frontal cortex to engage in higher-order thought processing [20]. Although it should be acknowledged that the extent of the differences concerning decision-making evaluation in the present research should be taken with some caution given the number of analyses conducted for the range of themes extracted, these findings suggest that players’ ability to engage in higher level thought processes may be reduced in hot conditions due to an even greater demand for resources in the primary motor cortex during more strenuous conditions.

Another possible explanation is that the heat stress in the present research could have also potentially induced some anxiety (as partially suggested by the negative affective states and higher threat state) that may have contributed in reducing soccer players’ focus during the decision-making task. For example, based on the premises of the Integrated Model of Anxiety and Perceptual-Motor Performance [63] anxiety can reduce focus on task-relevant cues and increase the likelihood of being distracted by task-irrelevant stimuli, and thereby possibly impaired the depth of decision-making in the heat. Future research could consider assessing specific emotions such as anxiety, rather than affective states, to test such possibilities. Additionally, the environmental heat stress may have contributed to a heightened demand for resources to process current events (i.e., maintaining power output and making the immediate decision), reducing athletes’ ability to predict and plan. This supports that fatigue induced by prolonged high-intensity intermittent exercise may result in athletes making decisions based on lower-level thought-processing [27], and that heat exposure may also impair higher-level appraisal of dynamic situations as they occur.

### Heat on physical performance, appraisal, affect and metanephrines

Heat also impaired athletes’ physical performance in this study. Power output was significantly lower during the second half compared to the first half in the hot condition (-~7%) but was not different between halves in the temperate condition. Previous research has shown that increased perceived exertion and more negative affective states induced by heat exposure can impair self-paced work rate [64]. Moreover, intermittent sprint performance is shown to be adversely affected by heat exposure, when marked hyperthermia is experienced (i.e., T_C_ >39°C) [65]. However, in this study, T_C_ reached values lower than those typically expected when performing team sport in the heat (>~39˚C) [65]. This was perhaps due to the inclusion of a cycling-based protocol, as metabolic heat production experienced during running based activity tends to be higher [66].

The present research also observed higher NMET and MET levels (measured as the more stable metabolites of norepinephrine and epinephrine) at half-time and post- exercise compared to pre-exercise, but such increases were to a much greater extent in the hot compared to temperate condition. This aligns with previous research that has observed that the release of epinephrine and norepinephrine is accelerated in response to acute heat stress [29]. The increased HR, fluid loss, RPE, thermal sensation as well as heightened threat and more unpleasant affective states, also observed in the heat, support that participants were under additional physiological and perceptual strain in the hot condition, despite T_C_ reaching average heights of ~38.4˚C. It is suggested that when players feel their T_C_ increasing more rapidly in hot conditions, they may deliberately or reflexively reduce their high-intensity work to reduce the rate of metabolic heat production and ensure thermal strain does not exceed tolerable limits [67]. In this study, unpleasant affective states were exacerbated toward the end of each half in the hot condition, which may have driven the reduction in work output observed from the outset of the second half. Core temperature was higher at the end of the first half compared to the second half in the hot condition, supporting that reducing high-intensity work reduced the levels of thermal strain experienced in the second half. This may also explain why decision-making performance did not decline more markedly in the second half in 32˚C. Nonetheless, such responses appear to support a heightened physiological and possibly psychological stress response in hot compared to temperate conditions.

The present study also identified that participants’ demand appraisals were higher and resource appraisals were lower in hot condition compared to the temperate condition, reflective of an overall higher threat state in the hot compared to the temperate condition. Naturally, when performing in hot environments, individuals are likely to perceive that higher effort may be required than in temperate conditions due to the widely understood exacerbated physiological responses (i.e., T_C_, HR sweat rate and dehydration) [22]. Furthermore, as competing in hot environments is uncommon for the sample in this study, some uncertainty as to how they would cope could be expected due to a lack of experience and knowledge in performing in these environments, which may have contributed to the heightened threat state observed [28]. Based on the premises of the TCTSA [28], threat states are argued to be linked with more unpleasant affective states, and impaired cognitive functioning (e.g., decision-making, concentration) and performance. Therefore, it is possible that heat exposure induced a higher threat state, which may have contributed to accounting for the more unpleasant affective states, impaired decision-making, and lowered physical work output, in the hot condition compared to the temperate condition in the present research. Although such causal inferences are beyond the scope of this research, further research may wish to consider directly testing this possibility.

### Limitations and future research

Whilst this study built upon previous methods by integrating a sport-specific visual decision-making task, research should incorporate tasks which require simultaneous visual and auditory attention (e.g., dual-task paradigms) to better replicate the demands of match-play. Additionally, a cycling intermittent sprint protocol was used to allow players to modulate their own high-intensity physical work output as per match-play, whilst simultaneously investigating cognitive function and central mechanisms. Despite this being widely employed within team sport literature [39,40], it is probable that physiological stress would have been more noticeable during running-based activity [66]. This would better replicate the demands of match-play and it is plausible that further cognitive decrements may be observed during running-based activity. Also, the premises of the TCTSA [28] is that challenge and threats states rely upon motivated performance. Conducting testing in a laboratory setting may have limited motivation compared to “real-world” competitive match-play. However, it could be assumed that these athletes were motivated due to them being well trained volunteer soccer players, and effort was incentivised through promotion of some level of competition simulated through the inclusion of a cash prize. Nevertheless, we do not know the extent that participants were motivated, nor understand the type of motivation they had, during the task. Therefore, researchers may wish to consider addressing such considerations in future studies to further enhance the external validity of our findings. It should also be noted that metabolites of noradrenaline and adrenaline were measured in this study, providing insights into MET and NMET activation during exercise in the heat. Researchers may also wish to measure cortisol and adrenocorticotropic hormone to assess PAC activation [e.g., 28] and provide deeper insights about neuroendocrinal response to exercise in the heat.

## Conclusions

In this research, soccer-specific decision-making was impaired in the hot compared to temperate condition. Physical work output was also significantly reduced in the second half in the heat but was not affected in the temperate condition. These decrements were observed when T_C_ was ≤38.5˚C, suggesting that complex, situation-specific decision-making as well as physical work output can be impaired below T_C_ thresholds proposed previously when exposed to heat during exercise [60]. Athletes also experienced a higher threat state and felt more unpleasant when exercising in the hot compared to the temperate condition, which could potentially have some role to play on the decision-making and physical work output decrements noted in the heat.

Soccer players and sports practitioners should consider that performing in more strenuous environments may enhance the likelihood of players making infrequent, but potentially costly, decision-making errors. Sports practitioners could look to reduce potential anticipatory effects of performing in more demanding environmental conditions by improving athletes’ perceptions of such environments, reducing their level of uncertainty, and improving their resource appraisal such as through enhancing self-efficacy and developing perceived control [28]. The implementation of a range of strategies could be considered (e.g., acclimatisation, cognitive or emotional-regulation strategies such as re-appraisal techniques) could help facilitate physical and decision-making performance during match-play in more demanding environments.

## Supporting information

S1 Table Fully counterbalanced results for the first eight participants. (DOCX)
