# Supplementary material for: The effect of exercise-induced fatigue and heat exposure on soccer-specific decision-making during high-intensity intermittent exercise
Source: PLoS One. 2022 Dec 15;17(12):e0279109. doi: 10.1371/journal.pone.0279109 (PMC9754236; doi:10.1371/journal.pone.0279109)
Supplement: S1 Table — (DOCX) [file pone.0279109.s001.docx]

|  | **Results with nine participants** | | | **Results with eight participants (fully counterbalanced)** | | |
| --- | --- | --- | --- | --- | --- | --- |
| **Variable** | **Condition** | **Time** | **Condition × Time** | **Condition** | **Time** | **Condition × Time** |
| **PPO** | *F*_1,8_ = 2.259, *p* = .171, η_p_^2^ = .220 | ***F*_1,8_ = 4.976, *p* = .056, η_p_^2^ = .383*** | ***F*_1,8_ = 6.659, *p* = .033, η_p_^2^ = .454*** | *F*_1,7_ = 1.821, *p* = .219, η_p_^2^ = .206 | *F*_1,7_ = 3.110, *p* = .121, η_p_^2^ = .308 | *F*_1,7_ = 4.816, *p* = .064, η_p_^2^ = .408 |
| **Decision-making quality** | ***F*_1,8_ = 6.930, *p* = .030, η_p_^2^ = .464*** | ***F*_2,16_ = 3.859, *p* = .043, η_p_^2^ = .325*** | *F*_2,16_ = 0.156, *p* = .857, η_p_^2^ = .019 | ***F*_1,7_ = 7.658, *p* = .028, η_p_^2^ = .522*** | *F*_2,14_ = 2.936, *p* = .086, η_p_^2^ = .295 | *F*_2,14_ = 0.155, *p* = .857, η_p_^2^ = .022 |
| **Decision-making lapses** | *F*_1,8_ = 4.414, *p* = .069, η_p_^2^ = .356 | *F*_2,16_ = 2.800, *p* = .086, η_p_^2^ = .264 | *F*_2,16_ = 2.800, *p* = .086, η_p_^2^ = .264 | ***F_1,7_* = 6.517, *p* = .038, η_p_^2^ = .482*** | *F*_2,14_ = 2.882, *p* = .089, η_p_^2^ = .292 | *F*_2,14_ = 2.882, *p* = .089, η_p_^2^ = .292 |
| **Core Temperature** | *F*_1,8_ = 1.531, *p* = .251, η_p_^2^ = .161 | ***F*_9,72_ = 16.142, *p* < .001, η_p_^2^ = .669*** | ***F*_9,72_ = 2.073, *p* = .043, η_p_^2^ = .206*** | *F*_1,7_ = 1.014, *p* = .348, η_p_^2^ = .127 | ***F*_9,63_ = 16.376, *p* < .001, η_p_^2^ = .701*** | *F*_9,63_ = 1.869, *p* = .073, η_p_^2^ = .211 |
| **HR** | ***F*_1,8_ = 13.581, *p* = .006, η_p_^2^ = .629*** | *F*_9,72_ = 33.384, *p* < .001, η_p_^2^ = .807 | ***F*_9,72_ = 3.375, *p* = .002, η_p_^2^ = .297*** | ***F*_1,7_ = 11.635, *p* = .011, η_p_^2^ = .624*** | ***F*_9,63_ = 26.954, *p* < .001, η_p_^2^ = .794*** | ***F*_9,63_ = 2.248, *p* = .030, η_p_^2^ = .243*** |
| **NMET^[[1]](#footnote-1)^** | ***F*_1,7_ = 43.637, *p* < .001, η_p_^2^ = .862*** | ***F*_1,7_ = 27.187, *p* < .001, η_p_^2^ = .795*** | ***F*_1,7_ = 7.278, *p* = .007, η_p_^2^ = .510*** | ***F*_1,6_ = 42.484, *p* = .001, η_p_^2^ = .876*** | ***F*_2,12_ = 24.742, *p* < .001, η_p_^2^ = .805*** | ***F*_2,12_ = 6.026, *p* = .015, η_p_^2^ = .501*** |
| **MET** | ***F*_1,7_ = 5.954, *p* = .045, η_p_^2^ = .460*** | ***F*_2,14_ = 37.670, *p* < .001, η_p_^2^ = .843*** | ***F*_2,14_ = 6.690, *p* = .009, η_p_^2^ = .489*** | ***F*_1,7_ = 5.954, *p* = .045, η_p_^2^ = .460*** | ***F*_2,14_ = 37.670, *p* < .001, η_p_^2^ = .843*** | ***F*_2,14_ = 6.690, *p* = .009, η_p_^2^ = .489*** |
| **Challenge/Threat** | ***F*_1,8_ = 13.069, *p* = .007, η_p_^2^ = .620*** | ***F*_8,64_ = 6.769, *p* < .001, η_p_^2^ = .458*** | *F*_8,64_ = 1.050, *p* = .409, η_p_^2^ = .116 | ***F*_1,7_ = 9.953, *p* = .016, η_p_^2^ = .587*** | ***F*_8,56_ = 5.049, *p* < .001, η_p_^2^ = .419*** | *F*_8,56_ = 1.116, *p* = .367, η_p_^2^ = .137 |
| **Affective Valence** | ***F*_1,8_ = 12.417, *p* = .008, η_p_^2^ = .608*** | ***F*_8,64_ = 12.159, *p* < .001, η_p_^2^ = .603*** | *F*_8,64_ = 1.991, *p* = .067, η_p_^2^ = .196 | ***F*_1,7_ = 11.527, *p* = .012, η_p_^2^ = .622*** | ***F*_8,56_ = 9.959, *p* < .001, η_p_^2^ = .587*** | *F*_8,56_ = 1.316, *p* = .255, η_p_^2^ = .158 |
| **RPE** | ***F*_1,8_ = 10.914, *p* = .011, η_p_^2^ = .577*** | ***F*_7,56_ = 20.451, *p* < .001, η_p_^2^ = .719*** | *F*_7,56_ = 1.155, *p* = .343, η_p_^2^ = .126 | ***F*_1,7_ = 8.754, *p* = .021, η_p_^2^ = .556*** | ***F*_7,49_ = 19.200, *p* < .001, η_p_^2^ = .773*** | *F*_7,49_ = 0.902, *p* = .513, η_p_^2^ = .114 |
| **TSS** | ***F*_1,8_ = 51.213, *p* < .001, η_p_^2^ = .865*** | ***F*_7,56_ = 9.101, *p* < .001, η_p_^2^ = .532*** | *F*_7,56_ = 1.403, *p* = .222, η_p_^2^ = .149 | ***F*_1,7_ = 39.79, *p* < .001, η_p_^2^ = .850*** | ***F*_7,49_ = 7.069, *p* < .001, η_p_^2^ = .502*** | *F*_7,49_ = 0.932, *p* = .491, η_p_^2^ = .117 |

1. ^1^Due to Participant 6 being removed from NMET/MET analysis, the original analysis was conducted on 8 participants, where 5 participants completed Trial 1 in 32˚C, and 3 participants completed Trial 1 in 18˚C. Therefore, follow up analysis for this variable was conducted on 7 participants, 4 of whom completed Trial 1 in 32˚C and Trial 2 in 18˚C. [↑](#footnote-ref-1)
